# Measurement of Compliance With New York City’s Regulations on Beverages, Physical Activity, and Screen Time in Early Child Care Centers

**DOI:** 10.5888/pcd11.130433

**Published:** 2014-10-16

**Authors:** Laura Lessard, Catherine Lesesne, Jakub Kakietek, Andrew Breck, Jan Jernigan, Lillian Dunn, Cathy Nonas, Sarah Abood O’Dell, Robert L. Stephens, Ye Xu, Laura Kettel Khan

**Affiliations:** Author Affiliations: Catherine Lesesne, Jakub Kakietek, Sarah Abood O’Dell, Robert L. Stephens, Ye Xu, ICF International, Atlanta, Georgia; Andrew Breck, National Foundation for the Centers for Disease Control and Prevention, Atlanta, Georgia; Jan Jernigan, Laura Kettel Khan, Centers for Disease Control and Prevention, Atlanta, Georgia; Lillian Dunn, New York City Department of Education, New York, New York; Cathy Nonas, New York City Department of Health and Mental Hygiene, New York, New York.

## Abstract

**Introduction:**

Policy interventions designed to change the nutrition environment and increase physical activity in child care centers are becoming more common, but an understanding of the implementation of these interventions is yet to be developed. The objective of this study was to explore the extent and consistency of compliance with a policy intervention designed to promote nutrition and physical activity among licensed child care centers in New York City.

**Methods:**

We used a multimethod cross-sectional approach and 2 independent components of data collection (Center Evaluation Component and Classroom Evaluation Component). The methods were designed to evaluate the impact of regulations on beverages served, physical activity, and screen time at child care centers. We calculated compliance scores for each evaluation component and each regulation and percentage agreement between compliance in the center and classroom components.

**Results:**

Compliance with certain requirements of the beverage regulations was high and fairly consistent between components, whereas compliance with the physical activity regulation varied according to the data collection component. Compliance with the regulation on amount and content of screen time was high and consistent.

**Conclusion:**

Compliance with the physical activity regulation may be a more fluid, day-to-day issue, whereas compliance with the regulations on beverages and television viewing may be easier to control at the center level. Multiple indicators over multiple time points may provide a more complete picture of compliance — especially in the assessment of compliance with physical activity policies.

## Introduction

About 24% of American children aged 0 to 4 years are enrolled in center-based child care and another 14% are cared for by a nonrelative adult (1). Thus, nearly 40% of young children spend most of their day being cared for by nonparent adults and are exposed to food and physical environments that are determined by their caregivers. The child care environment, including healthy nutrition and adequate physical activity, is an important factor in the health, well-being, and weight of young children. Many researchers and policy makers have suggested that health behavior patterns are established early in life, making the child care environment an essential element in national efforts to reduce childhood obesity and promote healthy behaviors even among the very young (2–4).

Policy can play a key role in regulating healthy child care environments; although research in this area is growing, increased evaluation of the effectiveness of such policies is needed (4,5). Once a policy is passed, there remain many ways in which the intended benefits of the policy may be compromised when it is implemented in real-world settings. It is critical to understand whether child care centers are able to comply with such policies.

This study examined group child care centers in New York City to assess whether and how well these centers comply with regulations, in place since 2007, intended to improve child nutrition, increase physical activity, and reduce noneducational screen time. New York City monitors compliance with regulations for licensed group child care centers using periodic (generally annual) site visits by city sanitarians; the assessment of compliance is basic, and the implications of poor compliance is an initial citation and a possible loss of license. To date, no center has lost its license because of the beverage, physical activity, and screen time regulations. Thus, our study sought to use a more comprehensive approach to assess compliance. Our approach, which measured compliance in different ways and in an applied research context, offered unique opportunities to investigate variability in compliance. Although recent research has shown relative validity of self-report and observational measures of child care nutrition and physical activity environments, additional evaluation is needed to identify reliable methods of assessing implementation of policies and regulations in real-world contexts (6).

## Methods

This evaluation used a multimethod cross-sectional approach and data collected from licensed group child care centers in New York City at 2 time points. This approach was designed to measure the level of compliance with regulations in centers and classrooms, the level of agreement in compliance between centers and classrooms, the factors that affect compliance, and the behavioral outcomes associated with different levels of compliance (eg, physical activity). Details on sampling and data collection are available in Breck et al (7), but they are explained briefly below.

### Sample

The sample was limited to centers serving low-income communities, defined by census tracts with 40% or more of families with incomes at or below 200% of the federal poverty level. The sampling frame included 300 of the 311 centers in District Public Health Office (DPHO) catchment areas (low-income, high-morbidity areas where increased levels of public health services are delivered). Ten centers in DPHO catchment areas were excluded because they were not in low-income census tracts; another center was excluded because it was in a census tract with fewer than 100 residents. An additional 350 centers in non-DPHO neighborhoods were included in the sampling frame. These centers were selected from among 549 centers in low-income non-DPHO census tracts in high-poverty zip codes. The goal was to obtain participation of 200 centers (approximately 12% of the licensed centers in New York City). To account for projected nonparticipation, centers were oversampled by 30% to create a sample of 260 centers by randomly sampling 130 DPHO centers and 130 non-DPHO centers. Selected centers were screened for participation eligibility (26 centers did not meet the eligibility criteria and were excluded from participation during recruitment). Of the 234 centers remaining, 58 refused and 176 agreed to participate in the evaluation. Most centers were located in the Bronx, Brooklyn, or Manhattan. Of the 176 centers that agreed to participate in the center component of the study, 110 (62.5%) also agreed to participate in the classroom component.

### Data collection

The study consisted of 2 data collections (the Center Evaluation Component and the Classroom Evaluation Component). The center component, conducted in fall 2009, focused on center-level data and included in-person interviews with each center’s director, 2 randomly selected teachers, and if applicable, a food service staff member. An observation of each center’s facilities, including kitchens and food items in pantries and refrigerators, was also conducted by trained site visitors. The classroom component, conducted in spring 2010, focused on classroom-level data and included observation of staff and child behaviors in the selected classroom during a 2-day site visit. The classroom was randomly selected by the data collectors if the center had more than 1 classroom. Trained data collectors observed all beverages and meals served and consumed, physical activity offered, screen time offered, and other characteristics in 1 classroom of children aged 3 or 4 years. They also collected data on physical activity via accelerometer (8). We measured compliance with 9 regulations and used the following data collection tools: in the center component, we used a site inventory, a food-service survey, a teacher survey, and a director survey; in the classroom component, we used a nutrition observation form, a mealtime observation form, and a general observation form (Table 1).

**Table 1:** Measures of Compliance for Center and Classroom Components for the Evaluation of New York City Regulations on Beverages, Physical Activity, and Screen Time for Group Early Child Care Centers (N = 110)

Regulation Component	Center Component (Fall 2009) Definition of Compliance by Source^a^	Classroom Component (Spring 2010) Definition of Compliance by Source
Serve only milk with 1% or less fat to children aged 2 years or older	Site inventory (n = 110): No milk with >1% fat was found in the center refrigerator.	Nutrition observation form: Data collector observed only unflavored milk with ≤1% fat served, or the center did not serve any milk.^b^
Provide only 100% fruit juice	Site inventory (n = 110): No <100% fruit juice was found in the refrigerator or on the shelf.	Nutrition observation form: Data collector observed only 100% fruit juice being served.
Serve no more than 6 oz of 100% fruit juice per day	Not measured.	Mealtime observation form^c^: Data collector recorded no more than 6 oz of 100% fruit juice was served to observed children (up to 6 children observed per center).
Do not serve beverages with added sweeteners, whether artificial or natural	Site inventory (n = 76): No beverages with added sweeteners were found in the refrigerator or on the shelf.	Nutrition observation form: Data collector observed no sugar-sweetened beverages served (including sweetened or flavored milk).
	Food service survey (n = 33): In an average week, staff reported never serving beverages with added sweeteners (eg, sodas, sports drinks, flavored or sweetened milk, Kool-Aid, Sunny Delight, Hawaiian Punch, lemonade, fruit drinks, *aguas frescas*, sweet tea) to the children.	
Make water available and accessible throughout the day, including at meals	Teacher survey (n = 105): In an average week, teaching staff reported making drinking water available to children all the time.^d^	General observation form: Data collector observed that drinking water was visible and accessible.
	Food service survey (n = 5): In an average week, food service staff reported making drinking water available to children all the time.	
Provide at least 30 min of structured physical activity per day	Teacher survey (n = 105): Teaching staff reported that children spend at least 30 total minutes per day in structured physical activity or movement time. “Structured” was defined as “teacher-led or teacher guided.”	General observation form: Data collector logged the start and stop times of all structured physical activity offerings. The difference between the start and stop time was used to calculate the amount of time for each structured physical activity event. Compliance was indicated when the summed time of all structured physical activity events was ≥30 min per day.
	Director survey (n = 2): Center director reported that children spend at least 30 total minutes per day in structured physical activity or movement time. “Structured” was defined as “teacher-led or teacher guided.”^e^	
Provide at least 60 min of total of physical activity per day, structured and unstructured combined	Teacher survey (n = 107): Teaching staff reported that children spend a combined total of at least 60 minutes per day of structured physical activity or movement time or unstructured or free play. “Unstructured” was defined as “times when the children are up and physically active, but the activity is not led by a teacher.”^af^	General observation form: Data collector logged the start and stop times of all unstructured physical activity offerings. The difference between the start and stop time was used to calculate the amount of time for each unstructured physical activity event. Compliance was indicated when the summed time of all structured and unstructured physical activity events was ≥60 min per day.
	Director survey (n = 2): Center director reported that children spend a combined total of at least 60 min per day of structured physical activity or movement time or unstructured or free play. “Unstructured” was defined as “times when the children are up and physically active, but the activity is not led by a teacher.”^g^	
Limit screen time to no more than 60 min per day	Teacher survey (n = 106): Teaching staff reported that in an average day, the children spend ≤60 min watching television or videos.	General observation form: Data collector recorded whether television was viewed or video/computer game playing was observed and for how many minutes each was observed. Compliance was indicated when the summed time of all television viewing and video/computer game playing was ≤60 min.
	Director survey (n = 2): Center director reported that in an average day, the children spend ≤60 min watching television or videos.	
Limit screen time viewing to educational programs or programs that actively engage child in movement^h^	Teacher survey (n = 107): Teaching staff reported that the children do not ever watch television shows or videos that are not for educational purposes.	General observation form: Data collector recorded that both the television and video/computer game viewing were for educational purposes only.
	Director survey (n = 2): Center director reported that the children do not ever watch television shows or videos that are not for educational purposes.	

a The first source listed is the primary source used to assess compliance status. When the primary source was not available, another source was used.

b Flavored or sweetened milk was considered a sugar-sweetened beverage.

c Mealtime observation form was used for mealtime observation of up to 6 children during 2 days, and the nutrition observation form was used for observation of food and beverage service (not consumption) in the classroom.

d Multiple teachers were asked this survey item, and the least compliant teacher response determined final compliance status; ie, if any teacher reported making drinking water available to children less than all the time, the center was deemed noncompliant.

e Half-day centers (n = 11) were deemed compliant when a respondent reported at least 15 minutes of structured physical activity or movement time.

f Multiple teachers were asked this survey item, and the least compliant teacher response determined final compliance status; ie, if any teacher reported that children spend less than 60 minutes in combined structured and unstructured physical activity, the center was deemed noncompliant.

g Half-day centers (n = 11) were deemed compliant when a respondent reported at least 30 minutes of structured physical activity or movement time.

h Programs that actively engage children in movement were not assessed in this study.

The evaluation was not originally designed to compare or validate measures of compliance with the regulations. Originally, the center component was designed to assess compliance at each center through the use of staff report and limited observation; the classroom component was designed to assess compliance-associated outcomes among children in selected classrooms. The center component was designed to be similar to regulatory compliance assessments typically conducted annually by government representatives. However, the classroom component was designed to use more resource-intensive methods — direct observation over a longer period of time — than those typically used for regulatory compliance assessments. The classroom component is less likely to be used for assessment of regulatory compliance in practice, but it could be used by center administrators to monitor their center’s compliance. 

Descriptive statistics were used to calculate compliance scores (as percentages) in both evaluation components. To explore the consistency of compliance with the regulations between the 2 components, we calculated the percentage of agreement between the components among the sample of 110 centers that had data from both components. All analyses were performed using SPSS version 19.0 for Windows (IBM Corp).

Although the purpose of the 2 data collections was not to compare or contrast the results of the 2 data collection methods used for assessing compliance, the results allowed our study team to document differences in compliance assessed by different methods. By examining differences in compliance measured by the 2 methods, our study elucidates strategies that can be applied to enforce these or similar regulations as well as implications for policy enforcement research.

## Results

In the center component, most child care centers were classified as compliant with the regulations on the type of milk to be served to children (80.0%), type of juice to be served (69.1%), the restriction on sugar-sweetened beverages (78.9%), water availability (89.1%), provision of at least 30 minutes of structured physical activity time (78.5%), total physical activity time of at least 60 minutes in a full day (87.2%), amount of television time permitted (100%), and provision of educational-only screen time (84.4%) (Table 2).

**Table 2:** Agreement Between Center and Classroom Component Compliance From the Evaluation of New York City Regulations on Beverages, Physical Activity, and Screen Time for Group Early Child Care Centers (N = 110)^a^

Regulation Component	Classroom Component Noncompliant, No. (%)	Classroom Component Compliant, No. (%)	Total, No. (%)	Agreement^b^, No. (%)
**1% Milk only**				
Center component noncompliant	7 (6.4)	15 (13.6)	22 (20.0)	91 (82.7)
Center component compliant	4 (3.4)	84 (76.4)	88 (80.0)	
Total	11 (10.0)	99 (90.0)	110 (100.0)	
**100% Juice only**				
Center component noncompliant	3 (2.7)	31 (28.2)	34 (30.9)	65 (59.1)
Center component compliant	14 (12.7)	62 (56.4)	76 (69.1)	
Total	17 (15.5)	93 (84.5)	110 (100.0)	
**Maximum of 6 oz of juice per day**				
Center component noncompliant	10 (9.1)	24 (21.8)	34 (30.9)	60 (54.5)
Center component compliant	26 (23.6)	50 (45.5)	76 (69.1)	
Total	36 (32.7)	74 (67.3)	110 (100.0)	
**No sugar-sweetened beverages (n = 109)**				
Center component noncompliant	4 (3.7)	19 (17.4)	23 (21.1)	77 (70.6)
Center component compliant	13 (11.9)	73 (67.0)	86 (78.9)	
Total	17 (15.6)	92 (84.4)	109 (100.0)	
**Water availability**				
Center component noncompliant	8 (7.3)	4 (3.6)	12 (10.9)	65 (59.1)
Center component compliant	41 (37.3)	57 (51.8)	98 (89.1)	
Total	49 (44.5)	61 (55.5)	110 (100.0)	
**Structured physical activity (n = 107)**				
Center component noncompliant	14 (13.1)	9 (8.4)	23 (21.5)	37 (34.6)
Center component compliant	61 (57.0)	23 (21.5)	84 (78.5)	
Total	75 (70.0)	32 (30.0)	107 (100.0)	
**Total physical activity (n = 109)**				
Center component noncompliant	9 (8.3)	5 (4.6)	14 (12.9)	42 (38.5)
Center component compliant	62 (56.9)	33 (30.3)	95 (87.2)	
Total	71 (65.1)	38 (34.9)	109 (100.0)	
**Television time (n = 108)**				
Center component noncompliant	0	0	0	93 (86.1)
Center component compliant	15 (13.9)	93 (86.1)	108 (100)	
Total	15 (13.9)	93 (86.1)	108 (100.0)	
**Television content (n = 109)**				
Center component noncompliant	2 (1.8)	15 (13.8)	17 (15.6)	84 (77.1)
Center component compliant	10 (9.2)	82 (75.2)	92 (84.4)	
Total	12 (11.0)	97 (89.0)	109 (100.0)	

a Unless otherwise indicated, the number of centers providing data was 110; data for constructing compliance scores for some regulations were missing for some centers.

b Percentage agreement was calculated by 1) adding together the number of centers that were compliant in the center component and the classroom component and the number of centers that were noncompliant in both components for a given regulation, and then 2) dividing the sum by the number of centers that provided data for both components.

Most centers in the classroom component were classified as compliant with type of milk served (90.0%), type of juice served (84.5%), restriction on sugar-sweetened beverages (84.4%), amount of television time permitted (86.1%), and provision of educational-only television time (89.0%). However, a smaller percentage of centers was classified as compliant in the classroom component with the amount of juice given to children (67.3%), water availability (55.5%), amount of structured physical activity offered (30.0%), and total amount of physical activity offered (34.9%). 

Compliance varied between components (Table 2). The percentage of centers that were classified as compliant in both the center and classroom components ranged from 21.5% (for structured physical activity) to 86.1% (for television time). We found a high percentage of agreement between center and classroom component compliance for milk (82.7%), sugar-sweetened beverages (70.6%), television time (86.1%), and provision of educational-only television programming (77.1%). In contrast, we found a low percentage of agreement between center and classroom component compliance for total physical activity time offered (38.5%) and structured physical activity time offered (34.6%).

## Discussion

Using different methods of assessment at 2 time points, we found high levels of reported and observed compliance with most regulation requirements. The percentage of centers that were classified as compliant in both components ranged from 21.5% (for structured physical activity) to 86.1% (for television time). Especially for television and milk, consistency between components was fairly high; centers that were classified as compliant in the center component were likely to be compliant in the classroom component about 4 to 6 months later. We hypothesize that the difference in compliance is because implementation of these requirements are easy to control at the center level, whereas the provision of physical activity is more sensitive to daily fluctuations, individual classroom factors (eg, variations among teachers or children), or other factors that make consistency of compliance more difficult to achieve. Kakietek et al (9) examined the factors that contributed to center compliance in this evaluation.

Each component of this evaluation used different data collection methods, and data were collected at 2 different times (about 4 to 6 months apart). The center component used self-reports and site observations, whereas the classroom component used a 2-day classroom observation in 1 randomly selected classroom. Although this evaluation was not intended to validate methods for assessing compliance with the regulations, it does shed light on the strengths and weaknesses of various methods of assessing regulatory compliance. For some policies, levels of compliance may vary across time; each day, a center and its staff must act to achieve compliance, and compliance with all regulation requirements may not always be achieved on any given day. Understanding compliance as a daily event might help to explain some of the variations found day to day and between center and classroom components.

For several regulation requirements, we found inconsistent compliance between the center and classroom components. One possible explanation for the inconsistency is that compliance with the regulations may have changed between the first and second data collections. Center component site visits were conducted in fall 2009, and the classroom component was conducted in spring 2010. Although the center staff members were not informed of the results of the center component, during the intervening time, changes in staff, facility, or other factors may have influenced the center’s implementation of the regulations.

A second possible explanation for the inconsistency in compliance is that data collected in the center component from teachers, directors, and food service staff members were self-reported inaccurately. This inaccuracy could have been caused by social desirability bias, inconsistent implementation within each center (eg, certain classrooms were compliant and others were not), or lack of knowledge among respondents about the practices or the policy or both. For example, although directors may have believed they served only 100% juice, they may have been unclear about the definition of 100% juice and unknowingly served juice drinks. Although the city provides training for center staff on the 100% juice policy, we do not know and did not assess in this study the extent to which the training results in comprehensive knowledge among center staff. The greatest difference in compliance between components was related to the provision of physical activity opportunities. Respondents in the center component may have wanted to provide the most socially desirable response: that they typically provide the required amount of physical activity opportunities. This explanation would account for the higher proportion of teachers who reported compliance in the center component than the proportion of teachers observed in the classroom component.

A third possible explanation for the inconsistency in compliance is measurement error. Although data collection tools were adapted from other studies where possible, our interview questions or observation tools may have incorrectly classified the center environment or staff behavior. Finally, differences in compliance between the center and classroom components may have resulted from atypical events on the days that data collectors visited the centers. In this scenario, data collected on an atypical day could result in higher or lower levels of compliance compared with data collected on a typical day.

The centers in our study were in low-income, urban neighborhoods; thus, generalizing our findings to other settings is cautioned. Given the resources involved in complying with these and similar regulations, higher-income neighborhoods could demonstrate higher levels of compliance than the centers participating in our study. However, comparisons of compliance assessed by the 2 different components used in our study may have applications for those monitoring compliance and those conducting applied research.

Another limitation of the study sample in both components is the potential for nonrandom bias in the rate of refusal to participate. Although centers included in the center component sample were selected randomly from a sampling frame, about 25% refused to participate in the center component, and about 38% of the centers who participated in the center component opted not to participate in the classroom component. Centers that took part in the classroom component were significantly more likely than centers that took part only in the center component to participate in the Child and Adult Care Food Program, be part of a larger parent agency, have dedicated food service staff, be in DPHO areas, and participate in training programs provided by the health department. Our data on refusal rates and characteristics of centers that refused to participate suggest that centers with poor compliance with the regulations were less likely to participate in the classroom component than centers with better compliance.

Although our evaluation was not designed to inform the enforcement of regulations, our methods may shed light on one of the practical difficulties of enforcing these regulations through the traditional means of an annual site visit by an inspector: the inability of inspectors to monitor compliance every day. Our study demonstrated on 2 separate occasions (1 additional observation per year than an inspector would make to each facility) that compliance with all components of the regulations varies over time and the method of assessing compliance may be especially important for physical activity requirements. We found a much lower level of compliance with the physical activity requirements when we used observation rather than director and teacher self-report. This lower level of compliance suggests that the policy’s intended benefit is less likely to be achieved. Policy makers may want to consider not only the content of such policies but also the mechanisms for ensuring compliance over time. For some policies, changes to the methods and frequency of compliance checks, penalties for noncompliance, and training and support may be needed to strengthen implementation.

Recent literature (6) suggests that self-reported survey responses, interviews, and observation are highly correlated for similar studies. Our study builds on that idea by combining multiple data sources to represent not only compliance for a single classroom at a single point in time but also center-wide compliance. Although our evaluation was not designed to validate measures of compliance, our methods can inform future research and evaluation. First, numerous measures could be used to assess compliance (eg, logs, other observational tools), although all methods have strengths and weaknesses. The measures used in our study were designed to capture data on classroom-level and center-level compliance at 2 time points to examine relationships between compliance and child-level outcomes (8). Second, researchers who are interested in validating compliance metrics would want to design studies explicitly focused on validation using multiple methods of assessment over time to draw more robust conclusions about the validity of individual methods. Although we could not verify which method was most reliable or valid, our findings suggest that observational methods may be a more conservative estimate of daily compliance with physical activity regulations. A study contrasting the use of logs to collect teacher-reported data on physical activity and the use of a third party to observe physical activity offerings would aid in identifying the most reliable, valid, and cost-efficient means of assessing compliance in group child care facilities.
